# Burnout syndrome in informal caregivers of older adults with dementia: A systematic review

**DOI:** 10.1590/1980-57642018dn13-040008

**Published:** 2019

**Authors:** Ludmyla Caroline de Souza Alves, Diana Quirino Monteiro, Sirlei Ricarte Bento, Vânia Diniz Hayashi, Lucas Nogueira de Carvalho Pelegrini, Francisco Assis Carvalho Vale

**Affiliations:** 1 Master’s student in Health Sciences, Graduate Program in Nursing, Federal University of São Carlos (UFSCar), São Carlos, SP, Brazil.; 2 PhD. Student in Health Sciences, Graduate Program in Nursing, Federal University of São Carlos (UFSCar), São Carlos, SP, Brazil.; 3 Nurse, São Carlos, SP, Brazil.; 4 PhD. Student in Sciences, Graduate Program in Fundamental Nursing, University of São Paulo (USP), Ribeirão Preto, SP, Brazil.; 5 Associate Professor, Department of Medicine, Federal University of São Carlos (UFSCar), São Carlos, SP, Brazil.

**Keywords:** caregivers, burnout, aged, dementia, stress, cuidadores, burnout, idoso, demência, estresse, sobrecarga

## Abstract

Dementia is a constant concern for the public health system. Also, it impacts family members who provide informal care, often culminating in overload due to the emotional stress and physical distress of caregivers.

**Objective:**

This systematic review aimed to identify the consequences of Burnout Syndrome in informal caregivers of older adults with dementia.

**Methods:**

The search was performed spanning the last 10 years, in English, Portuguese or Spanish. The databases used were PubMed, SciELO, Web of Science and LILACS. The descriptors were obtained from MeSH and DeCS, which were, “caregivers”, “burnout”, “aged”, “psychological stress” and “dementia”. The selected articles included studies conducted with informal caregivers of community-dwelling older adults diagnosed with any type of dementia. The excluded articles had the following characteristics: the participants were not informal caregivers, the older adults were not diagnosed with dementia, or the main theme was not related to the Burnout Syndrome.

**Results:**

Initially, 1,208 articles were found. One hundred and forty-six were eliminated because they were duplicates. A further 1,033 were excluded because they did not meet the inclusion criteria. Twenty-nine studies were selected for full reading and 22 were excluded, giving 7 studies for inclusion in this review.

**Conclusion:**

The results showed that the Burnout Syndrome negatively affected caregivers’ quality of life and was associated with patient depressive and anxious symptoms and abusive behavior by caregiver. There is a need for studies with interventions addressing this issue.

## RESUMO

A demência é uma preocupação constante para o sistema público de saúde. Além disso, traz impactos para os familiares que oferecem cuidados informais, culminando frequentemente em sobrecarga, devido ao estresse emocional e desgaste físico dos cuidadores.

**Objetivo:**

Esta revisão sistemática objetivou identificar as consequências da Síndrome de Burnout em cuidadores informais de idosos com demência.

**Métodos:**

A busca foi feita considerando-se os últimos 10 anos, nos idiomas inglês, português ou espanhol. As bases de dados utilizadas foram PubMed, SciELO, Web of Science e LILACS. Os descritores foram obtidos no MeSH e DeCS, sendo “cuidadores”, “burnout”, “idoso”, “estresse psicológico” e “demência”. Os artigos selecionados incluíram estudos desenvolvidos com cuidadores informais de idosos que vivem na comunidade, diagnosticados com qualquer tipo de demência. Os artigos excluídos apresentaram as seguintes características: os participantes não eram cuidadores informais, os idosos não receberam diagnóstico de demência, ou o tema principal não estava relacionado à Síndrome de Burnout.

**Resultados:**

Inicialmente, foram encontrados 1.208 artigos. Destes, 146 foram removidos por serem duplicados. Após, 1.033 foram excluídos por não atenderem aos critérios de inclusão. Assim, 29 estudos foram selecionados para leitura na íntegra e 22 foram excluídos. Finalmente, 7 estudos foram selecionados para esta revisão.

**Conclusão:**

De acordo com os resultados, foi possível identificar que a Síndrome de Burnout afeta negativamente a qualidade de vida dos cuidadores e está associada a sintomas depressivos, ansiosos e comportamento abusivo. Há a necessidade de estudos com intervenções que abordem esta temática.

Dementia has become a public health issue worldwide. Its annual economic impact is about one trillion dollars and growing.^1^ This is due to the high number of individuals living with dementia in the world, which is 50 million. Projections suggest this figure will increase three-fold by 2050, when the number of patients with dementia is set to reach 152 million.^1^ Also, most of these older adults with dementia will be cared for by family or community members.^2^^,^ 3

The number of caregivers has increased considerably.^4^ Caring for others can be beneficial; however, research has suggested a higher prevalence of overload developed by emotional stress, physical wear, limited social/leisure activities, and lack of appetite and sleep, as well as an increased risk of mortality and mental disorders in caregivers compared with non-caregivers.^2^^,^ 5 ^-^ 7

Informal care involves a multitude of complex activities provided by non-professional carers.^4^^,^ 8 These carers can be family members, close relatives, friends, or even neighbors. Their goal is to assist older adults who have reduced autonomy and independence in the execution of activities of daily life.^7^^,^ 8 In addition, informal caregivers neither have an established working time nor receive payment for their efforts.^7^^,^ 9 Also, they are usually emotionally involved with the person being cared for.^3^^,^ 4 ^,^ 8 ^,^ 10

Caregiving load can be divided into two: objective load and subjective load.^3^^,^ 11 The first refers to the economic impact and time spent on caring activities; the other refers to caregivers’ emotional responses.^3^^,^ 11 ^,^ 12 Accordingly, the concept of burnout refers to the association between stress and caring load, as well as with critical aspects that caregivers establish with their occupation.^3^

Although research involving burnout was originally carried out in workers from social and health fields, the number of studies that investigate burnout in informal caregivers has been rising in the literature.^3^ In addition, studies have suggested that family caregivers of older adults with dementia may also suffer from burnout.^13^^,^ 16 Social isolation, poor health, and negative perspective on caring represent important burnout predictors among family caregivers of elderly with dementia.^11^^,^ 14

The overload observed in informal care has implications for caregiver health, which reduces the quality of care.^17^ A recent literature review shows that Burnout Syndrome among family caregivers is due to patients’ limitations.^18^ It states that the greater the limitation, the higher the overload.^18^

Burnout Syndrome is characterized by a psychosocial syndrome that arises in response to chronic and interpersonal stressors in the workplace. However, it is important to note that this concept is not only related to caregiving load, but also to critical aspects of relationships that the person establishes with his/her occupation.^19^ This syndrome is characterized by three dimensions: emotional exhaustion (lack of energy and enthusiasm, as well as no emotional resources), depersonalization (adoption of an indifferent, impersonal and even cynical attitude between caregiver and patient), and reduction of personal fulfillment (perceiving care as negative or ineffective).^19^^,^ 20

The effects and consequences of Burnout Syndrome may develop into two main situations: mental (low self-esteem, exhaustion, anxiety, frustration, lack of concentration, and clinical manifestations such as headaches, insomnia, pain and gastrointestinal problems) and behavioral (caffeine consumption, tranquilizers, and licit drugs).^21^ Some factors may interfere in the quality of care and lead to early patient institutionalization, as well as to social isolation, long drawn out stress, and biological vulnerability, which may increase the risk of mental and physical problems for caregivers (e.g. hypertension, increased stress-related hormones, suppressed immunity, major depression, and exhaustion).^22^

Therefore, considering the lack of studies about the factors associated with burnout in informal caregivers, as well as the fact that caregiver burden affects caregivers’ quality of life and quality of care, our research question is: how does burnout affect the care provided by informal caregivers to older adults with dementia? 

This systematic review aimed to identify studies about Burnout Syndrome among informal caregivers of elderly with dementia, and how it affects care. Furthermore, the implications of the syndrome in this group were also explored.

## METHODS

A systematic literature review spanning the past 10 years was conducted to select studies whose topics were about Burnout Syndrome in informal caregivers of the elderly with dementia. The search was conducted in October/2018 on the following databases: PubMed, LILACS (Literatura Latino-americana e do Caribe em Ciências da Saúde), SciELO (Scientific Electronic Library Online), and Web of Science. Descriptors were obtained in DeCS (Descritores em Ciência da Saúde) and MeSH (Medical Subject Headings) and were: caregivers, stress, burnout, aged, and dementia, as well as their correlates in Portuguese and Spanish.

Search strategies were based on the above-mentioned descriptors. The Boolean operator “AND” was used for the following combinations: “Cuidadores AND Burnout”; “Caregivers AND Burnout”; “Cuidadores AND Burnout AND Estresse Psicológico”; “Caregivers AND Burnout AND Stress”; “Cuidadores AND Burnout AND Estrés Psicológico”; “Cuidadores AND Burnout AND Idosos”; “Caregivers AND Burnout AND Aged”; “Cuidadores AND Burnout AND Anciano”; “Cuidadores AND Burnout AND Demência”; “Caregivers AND Burnout AND Dementia”; “Cuidadores AND Burnout AND Demencia”.

Some filters were applied in order to refine the search: articles published between 2008 and 2018; in Portuguese, Spanish, and English. In PubMed and Web of Science, the required document was article, and the search was for title and abstract. In SciELO, the search was by title; in LILACS, articles were searched by all fields (i.e. title, author, and topic).

Inclusion criteria were: publication date in the last 10 years; articles in English, Spanish, and Portuguese; studies whose topic was Burnout Syndrome in informal caregivers of older adults with dementia; and availability (access possible). Articles were excluded if they did not focus on caregivers of elderly, informal caregivers, or caregivers of older adults with dementia, and studies whose topic was not Burnout Syndrome. Papers about caregivers of older adults living in nursing homes or literature reviews, theses, dissertations, and monographies were also excluded.

Preferred Reporting Items for Systematic Review and Meta-Analysis (PRISMA) was used for the article selection process. PRISMA aims to help authors improving communication on systematic reviews and meta-analysis, and is also used for critical evaluations of previously published systematic reviews.^23^ Regarding data analysis and extraction, an adapted version of an instrument proposed by Ursi (2005) was used.

## RESULTS

**Figure 1:**
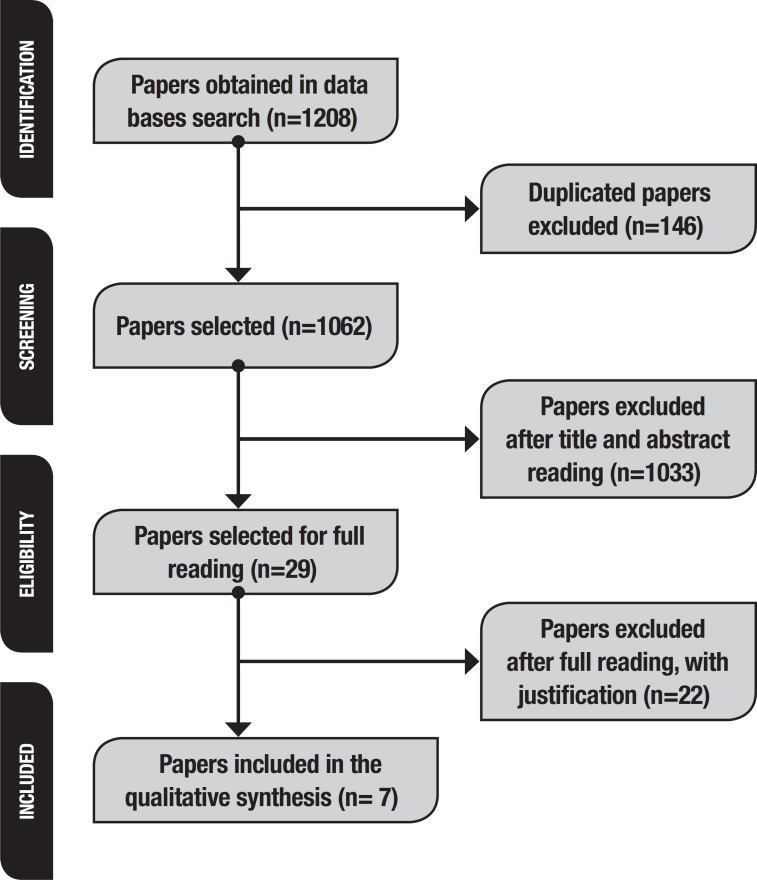
Illustrative Summary of Paper Selection process for the Systematic Review (PRISMA) Method, São Carlos, SP, Brazil, 2018.

The search of the databases resulted in the retrieval of 1,208 articles. Of these, 146 were eliminated due to duplication. In the next step, 1,033 studies were excluded after title/abstract reading because they did not meet the inclusion criteria. Subsequently, 29 papers were selected for full reading, and 22 were excluded after this process, giving 7 studies for inclusion in this systematic review. Figure 1 shows a schematic representation of paper selection according to the PRISMA method.

Of the obtained articles, two were conducted in Rio de Janeiro, Brazil.^16^^,^ 22 Regarding age group, only four articles mentioned this information and in two of these participant mean age was 60 years or older.^16^^,^ 24 All studies had a quantitative design and most were cross-sectional.

In addition to Burnout Syndrome, five studies evaluated depressive symptoms, anxiety, and quality of life. One article analyzed the relationship between Burnout Syndrome and domestic violence driven by the occupational status of being a caregiver of an older adult with dementia.^25^ The other study evaluated ways of dealing with the elderly.^15^ Four studies used the MBI, and three used the Pines Burnout Measure (PBM) to evaluate Burnout Syndrome in the population studied.

Table 1 shows information obtained from the studies selected for this systematic review.

**Table 1:** Burnout syndrome studies in caregivers of older adults with dementia.

Author/ Place	Demographics	Design	Measuring Instrument	Main findings
Ylmaz et al., 2009 Turkey	N = 44 Mean age: 61.6 (±10.4)	Quantitative Cross-sectional	• MBI • Sociodemographic questionnaire • HAM-D • HAM-A • WCQ	A correlation between patient depression level and caregiver depersonalization was found.
Takai et al., 2009 Japan	N = 84 Mean age: 59.5 (±13.9)	Quantitative Cross-sectional	• PBM • BDI • WHOQOL	Higher levels of burnout were correlated to higher depressive symptoms and less quality of life. Results suggest interventions/strategies to improve the analyzed variables.
Valente et al., 2011 Brazil	N = 137 Mean age: 56.8 (±14.0)	Quantitative Cross-sectional	• MBI • Sociodemographic questionnaire • BDI • BAI • ZBI	Anxiety symptoms and physical problems are risk factors for poor self-perceived health of caregiver.
Takai et al., 2011 Japan	N = 118 Mean age: 60.9 ( ±14.0)	Quantitative Cross-sectional	• PBM • WHOQOL-26 • BDI	Depressive symptoms and burnout were associated with worse quality of life for caregivers.
Truzzi et al., 2012 Brazil	N = 145 Mean age = 76.4 (±6.9) Time as caregiver = 3.8 (±2.7)	Quantitative Cross-sectional	• MBI • Sociodemographic questionnaire • BAI • BDI	Caregiver depression and patient *delirium* were the main factors predicting emotional exhaustion.
Yan, 2014 Hong Kong	N = 149	Quantitative Prospective	• MBI • CMAI • CTS2	Older adult restless behavior and caregiver burnout symptoms were associated with abusive behavior by family caregiver.
Hiyoshi-Taniguchi, 2018 Japan	N = 80	Quantitative Cross-sectional	• NPI-D • PBM	No relationship found between burnout symptoms and apathy, depression or anxiety.

MBI: Maslach Burnout Inventory; HAM-D: Hamilton Depression Rating Scale; HAM-A: Hamilton Anxiety Rating Scale; WCQ: Ways of Coping Questionnaire; PBM: Pines Burnout Measure; BDI: Beck Depression Inventory; WHOQOL: World Health Organization Quality of Life; BAI: Beck Anxiety Inventory; ZBI: Zarit Burden Interview; CMAI: Cohen-Mansfield Agitation Inventory; CTS2: Revised Conflict Tactics Scales; NPI-D: Neuropsychiatric Inventory Caregiver Distress Scale.

## DISCUSSION

The goal of this study was to find in the literature studies assessing Burnout Syndrome in informal caregivers of elderly with dementia, and also investigating the impact of this syndrome on different aspects of care and caregivers’ lives.

Regarding participants’ sociodemographic characteristics, all studies showed a higher prevalence of female caregivers, most of whom were the patient´s daughter. The exception was a study^24^ in which the main caregiver was the spouse. These findings corroborate the literature, for example, the study conducted by Da Silva et al.,^26^ in which the authors evaluated the relationship between care and overload in informal caregivers. In the study, of 58 participants, 42 (72.7%) were adult daughters.^26^ According to the authors, this is a matter of family hierarchy, in other words, when wives are unable to provide care, older daughters assume this role.^1^^,^ 26

In addition five studies involved middle-aged adults (≥50 years old). In a study about repercussions of care in the life of family caregivers of older adults with Alzheimer’s disease, a wide variation in age range was observed (between 40 and 60 years old).^27^ Even though in both studies elderly caregivers were not as prevalent, it is important to observe that these individuals are almost reaching senescence and thus will probably be older adults taking care of older adults and might themselves need support.

Of the seven studies, six described participants’ educational level. In five studies, participants had completed high school or held an undergraduate degree. In one study,^25^ only 28 (18%) caregivers out of a total of 149, had finished high school. Educational levels >8 years were also found in research that evaluated overload and Burnout Syndrome in informal caregivers of older adults with dementia.^26^^-^ 28 One study did not mention participants’ years of education and was conducted in family caregivers from urban and rural areas of Japan.^29^

Educational background is an important variable to be studied because it demonstrates the level of knowledge about the care provided by caregivers, which may contribute toward understanding the disease and learning better ways of dealing with the process.^9^^,^ 30 In this context, it is relevant to highlight the importance of training for caregivers. Of the analyzed studies, only one mentioned this topic, showing that, out of the total 149 participants, 29 received training on caring for a family member.^25^

Regarding the type of dementia, two studies did not specify the diagnosis.^25^^,^ 29 The other investigations cited the following types: Alzheimer’s Disease, vascular dementia, frontotemporal dementia, mixed dementia, and Lewy Body dementia. One study^15^ specifically evaluated Alzheimer’s Disease alone, while in two studies^16^^,^ 31 the diagnoses were Alzheimer’s Disease, vascular dementia, and mixed dementia. Finally, the other two studies^9^^,^ 24 observed all types mentioned above. Among those articles that evaluated dementia, Alzheimer’s Disease was the most prevalent cause. The same pattern was observed in a study^11^ analyzing the existence of a relationship between dementia subtype and self-perceived health of caregivers. In the study, 22% (N=49) of patients were diagnosed with Alzheimer’s Disease.

Another variable of interest in this study was older adults’ level of dementia. Unfortunately, none of the studies expanded on this information, only mentioning that the patients were diagnosed and followed by specialized doctors. 

As a consequence of dementia, behavioral symptoms may be common in older adults, and manifest according to the disease characteristics. All the analyzed studies identified older adults’ behavioral symptoms, such as agitation, apathy, aggressiveness, depression, and others. Also, symptoms like depression, anxiety and hopelessness were observed in caregivers, and were associated with Burnout Syndrome. 

The screening for Burnout Syndrome is usually conducted using instruments. All of the analyzed studies used this strategy. The most used instrument was the Maslach Burnout Inventory (MBI), followed by the Pines Burnout Measure (PBM), employed in three studies - two by the same author.^14^^,^ 24 The literature also indicates the MBI and PBM as the most common instruments for analyzing Burnout Syndrome. The MBI is a 22-item questionnaire whose questions are related to Burnout’s three dimensions. The answers are based on a Likert scale ranging from 1 (totally disagree) to 7 (totally agree). The PBM is a 21-item self-reported questionnaire that evaluates physical and emotional aspects, as well as mental exhaustion.^26^

Regarding burnout symptoms, in two articles, caregiver depersonalization was a prevalent symptom.^15^^,^ 25 The first article related it to patient depression, and the second, to patient restless behavior. In two other studies, emotional exhaustion was more prevalent. In their study, Valente et al. found emotional exhaustion to be more expressed in caregivers of older adults with moderate dementia, which led to a negative self-perception of health.^31^ In addition, Truzzi et al. showed that high levels of emotional exhaustion are related to patient *delirium* and caregiver depression, in other words, the authors suggested that both variables are related to this dimension (emotional exhaustion).^16^ In a different study, the same authors found emotional exhaustion to be more prevalent, followed by depersonalization.^26^ Furthermore, emotional exhaustion was closely related to anxiety and depressive symptoms,^14^ which was also observed in the two studies cited above.

Regarding the results obtained by Takai et al.,^14^^,^ 24 in their two studies using the PBM, the most prevalent group was the “without burnout” group. It is important to mention that the PBM classifies participants according to their scores into four groups as follows: without burnout (≤2.9), at risk of developing burnout (3 - 3.9), present burnout (4 - 4.9), and clinically depressed (≥5). Although the number of participants without burnout was bigger in these studies,^14^^,^ 24 a study^14^ of 84 caregivers investigated the relationship between burnout, depression, and quality of life, revealing that 19% of caregivers had burnout and 22.6% were at risk of developing the syndrome.

Similarly, Hiyoshi-Taniguchi et al. used the same scale to identify which behaviors of patients with dementia contributed to the onset of Burnout Syndrome in the caregiver.^29^ In their study, caregivers mentioned high levels of anguish, which was related to patient behavioral symptoms, but their scores on the Pines scale were low.^29^ On the other hand, the same study showed that symptoms like agitation and aggression, irritability, abnormal motor behavior, and hallucinations in patients with dementia led to high scores for Burnout Syndrome.^29^

Therefore, these studies showed that Burnout Syndrome was present in informal caregivers of older adults with dementia. Also, the syndrome was related to depression and anxiety symptoms, patients’ behavioral symptoms, and clinical and sociodemographic factors, for example. In general, the authors of the analyzed studies suggested longitudinal studies involving larger samples. They also suggested the need for early detection of Burnout Syndrome, in other words, screening should identify groups at risk. Regarding interventions, the authors recommended that health professionals be aware of caregivers’ needs.

A limitation of this systematic review was the exclusion of articles that addressed Burnout Syndrome both in formal and informal caregivers of older adults with dementia, but did not separate them into groups. Consequently, it was not possible to analyze only results related to informal caregivers in these studies. Also, finding studies that addressed a deeper approach to Burnout Syndrome (e.g. showing the percentage/proportion of participants in each syndrome’s dimension) was difficult. This could be helpful for proposing assertive intervention strategies. Another limitation pertains to the fact that the use of the concept “burnout” was originally proposed for formal occupations. Despite this, studies have investigated this condition for informal workers (e.g. informal caregivers), which makes it possible to think about the need of adapting this concept to the context of different occupations.^32^

The selected studies mentioned the importance of evaluating Burnout Syndrome in informal caregivers due to the relationship with worse quality of life, depressive symptoms, anxiety, and abusive behavior among caregivers. Furthermore, articles suggested that interventions and strategies focused on this group may help to improve these symptoms. However, studies dedicated to this topic (i.e. Burnout Syndrome in informal caregivers of older adults with dementia) are scarce, hence more studies about this issue are necessary.^32^
